# Memory for Expectation-Violating Concepts: The Effects of Agents and Cultural Familiarity

**DOI:** 10.1371/journal.pone.0090684

**Published:** 2014-04-08

**Authors:** Michaela Porubanova, Daniel Joel Shaw, Ryan McKay, Dimitris Xygalatas

**Affiliations:** 1 Department of Psychology, Farmingdale State College, Farmingdale, New York, United States of America; 2 Laboratory for the Experimental Study of Religion, Masaryk University, Brno, Czech Republic; 3 Social and Behavioural Neuroscience Research Group, Central European Institute of Technology, Masaryk University, Brno, Czech Republic; 4 ARC Centre of Excellence in Cognition and its Disorders, and Department of Psychology, Royal Holloway, University of London, London, United Kingdom; 5 Interacting Minds Centre, Department of Culture and Society, Aarhus University, Aarhus, Denmark; Durham University, United Kingdom

## Abstract

Previous research has shown that ideas which violate our expectations, such as schema-inconsistent concepts, enjoy privileged status in terms of memorability. In our study, memory for concepts that violate cultural (cultural schema-level) expectations (e.g., “illiterate teacher”, “wooden bottle”, or “thorny grass”) versus domain-level (ontological) expectations (e.g., “speaking cat”, “jumping maple”, or “melting teacher”) was examined. Concepts that violate cultural expectations, or counter-schematic, were remembered to a greater extent compared with concepts that violate ontological expectations and with intuitive concepts (e.g., “galloping pony”, “drying orchid”, or “convertible car”), in both immediate recall, and delayed recognition tests. Importantly, concepts related to agents showed a memory advantage over concepts not pertaining to agents, but this was true only for expectation-violating concepts. Our results imply that intuitive, everyday concepts are equally attractive and memorable regardless of the presence or absence of agents. However, concepts that violate our expectations (cultural-schema or domain-level) are more memorable when pertaining to agents (humans and animals) than to non-agents (plants or objects/artifacts). We conclude that due to their evolutionary salience, cultural ideas which combine expectancy violations and the involvement of an agent are especially memorable and thus have an enhanced probability of being successfully propagated.

## Introduction

Research in psychology and anthropology has looked into the question of what makes some ideas more culturally successful than others. It has been suggested that cultural ideas enjoy a cultural transmission advantage because they appeal to human cognitive architecture [1], [2], [3], [4], [5], [6]. The success of an idea is determined by psychological factors, such as how attention is attracted to a particular idea, and subsequently, how easily this idea is represented and remembered [4], [7], [8].

In memory research, it has been suggested that items inconsistent with our expectations are recalled better than those consistent with our expectations [9], [10], [11], [12], [13], [14], a phenomenon often referred to as the “von Restorff effect” [15], or the “distinctiveness effect”. Similarly, concepts violating our ontological expectations seem to have a unique position in attracting attention and leading to distinct encoding. Yet, research in the area of cognition and culture has predominantly focused on domain-level breaches, i.e., breaches in intuitive ontologies, with the exception of several studies [16], [17], [18], [19]. However, the role of agents in memorability to conceptual information has been largely overlooked. Throughout their ontogeny, humans become attuned to understanding how actions and events in the world operate and what can be expected or unexpected yet viable, i.e., they acquire intuitive ontological assumptions [20], [21], as well as assumptions about who can perform those actions. When presented with information, humans activate the potential characteristics of that information and compare the incoming information with already existing knowledge [22], making inferences about possible outcomes of events and their respective probabilities and also activating a set of expectations employed when encountering new information [23], [24]. Furthermore, the human mind is endowed with core knowledge systems, which provide general inferences about various domains (objects, actions, numbers, space), and new knowledge is generated based on the foundations of those core systems [25].

Focusing on the domain of religious ideas, Pascal Boyer [26] introduced the notion of a “cognitive optimum” – a balance between attention and cognitive effort. He argued that minimally counterintuitive (MCI) concepts (those that include a limited number of domain-level violations, such as “flying cat” or “stalking chair”) are more likely to be remembered, and thus more successfully transmitted. Such concepts infringe upon some fundamental assumptions about domain-specific knowledge, like intuitive psychology (theory of mind), biology, or physics [27], [28]. This particular feature gives those ideas powerful inferential potential, allowing for various inferences and interpretations, resulting in their easier representation and memorability.

Experimental studies have suggested that the presence of mild violations of intuitive ontological expectations in either concept form or narrative material is optimal for human attention and memory, and thus beneficial for the transmission of those concepts [1], [2], [5], [29]. However, other studies suggest a more complex picture, showing that context might play a more important role in the memorability of individual concepts [30], [31], [32], [33], [34].

This variation in the results of the studies can be attributed possibly to using different kinds of material (either at a narrative or concept level), to the use of uncontrolled confounding variables such as word length and word frequency, and/or to an uncontrolled exposure duration of individual concepts. Importantly, processing time, which is a particularly important factor both for attention and memory, was not controlled for in the majority of extant studies as participants were permitted to spend various amounts of time reading and studying concepts embedded in stories [1], [30], [34], [35] or concept lists [5], [30]. This variability in processing time might have resulted in attentional preference for more unique, slightly bizarre concepts (such as minimally counterintuitive concepts), with factors such as post-stimulus elaboration making the memory trace less prone to forgetting [36]. Only a few studies have controlled for confounding factors such as processing time, word frequency and word length [31], [37], [38]. Our study offers a precise examination of memory for expectancy-violating concepts while controlling for these confounding variables.

An additional factor that might bias the memorability of concepts with violations to either domain-level or cultural schema-level expectations is the presence of agents in the individual concepts. A prominent attribute of religious ideas is that many of them contain minimally counter-intuitive concepts in which non-agents are ascribed with agent-like qualities (e.g., a whispering rock). Moreover, another aspect of religious ideas is their “stickiness”, due to the inclusion of agents possessing supernatural qualities. The human propensity for detecting agents in ambiguous situations is unavoidable and powerful [39], and has been related to adaptive mechanisms that facilitate the identification of potentially harmful agents [40], [41]. It has been proposed that humans have developed a sensitive hazard-precaution system to defend themselves against potential dangers (predation, contagion, intrusion by strangers etc.) [42]. Intentional agents in particular might often represent a potential threat and therefore it is extremely advantageous to be able to detect them effectively. Thus, overattribution of agents, even when there is none [43], [44], might be a beneficial strategy for survival.

Based on the above, memory for agents (i.e. humans and animals) should be stronger compared to memory for non-agents (e.g., plants and objects) across all concept categories. Therefore, examining memory for cultural schema-level versus domain-level violations, each as related to agents versus non-agents, provides an important addition to the current research on understanding the memorability of cultural ideas.

Here, we present two experiments tapping into this problem by using immediate recall as well as surprise delayed recognition tasks. Both experiments were aimed at examining how ideas, in our case concepts, entertain human memory according to the type of expectation violation as well as the involvement of agents or non-agents. Intuitive, schema-consistent information does not violate any expectations: the concepts represent information that can be encountered in the real world. Schema refers to the employment of simplified, shared cultural knowledge that helps predict and anticipate events, agents, and actions [41] by representing their prototypical attributes that are available in a specific situation [42]. Furthermore, we introduced two different expectation violations. First, ontological (or domain-level) expectation-violating ideas, i.e. ideas breaching intuitive ontologies; and second, counter-schematic (or cultural schema-level expectation violating) concepts- i.e. those violating culturally shared knowledge while retaining intuitive expectations. Introducing these novel factors in the study of concept memorability can potentially elucidate some of the mechanisms underlying the success of certain cultural ideas.

## Methods

### Ethics statement

Written informed consent was obtained from all participants and the study was approved by the ethics committee of the Czech Association for the Study of Religions.

### Immediate memory recall

Our first experiment investigated the role of expectation violation on subsequent memory recall. We used a simple task in which participants were presented with concepts randomly from three different concept categories. Two categories pertained to expectancy-violating concepts. The first category included ontological violations (ONT) which are claimed to be an important and underlying component of the success of many cultural ideas [26]. These ideas violate intuitive ontologies, i.e., information pertaining to the domains of folk physics (e.g., an object falling upwards), folk biology (e.g., a singing tree), and folk psychology (e.g., a person that can predict the future). The second category was represented by concepts that violate cultural schema-level expectations, i.e., common or prototypical attributes of information stored in the conceptual knowledge system. In other words, they violate cultural intuitions and refer to cultural schema-level breaches (CUL) (e.g., an illiterate teacher or a wooden bottle), while ontological intuitions are maintained. Cultural schema-level breaches comply with expectations pertaining to folk biology, folk psychology, and folk physics, but violate expectations related to culturally acquired schemas. Finally, the third category was represented by intuitive concepts (INT), i.e., concepts that refer to everyday, mundane concepts, agents, and objects that do not breach any expectations (e.g., a green pencil, a smart chemist, etc.). The aim was to examine memory for individual concept categories. The individual concepts used in the study are displayed in Table 1.

**Table 1:** Individual concepts pertaining to cultural schema-level violations, ontological violations, or intuitive ideas.

	Concept Category		
Ontological Category	CUL	ONT	INT
**Human**	Atheist priest	Flying waiter	Classy artist
	Blind driver	Liquid butcher	Honest writer
	Illiterate teacher	Melting teacher	Salivating runner
	Puritan whore	Transparent pilot	Smart chemist
**Animal**	Carnivorous sheep	Democratic skunk	Drinking dog
	Coward tiger	Evaporating rabbit	Galloping pony
	Domestic bear	Speaking cat	Obedient horse
	Herbivorous hyena	Swearing koala	Tame zebra
**Plant**	Salty banana	Barking grape	Drying orchid
	Soft cactus	Jumping maple	Green hedge
	Stinky rose	Racing tulip	Growing pine
	Thorny grass	Vomiting birch	Planted onion
**Object**	Spherical room	Hungry kettle	Brown fence
	Stone mirror	Stalking table	Convertible car
	Triangle plate	Talking train	Green pencil
	Wooden bottle	Worried chair	Plastic clock

### Subjects

Seventy-five undergraduate students (32 male and 43 female) volunteered for the study. A total of 70 participants finished both sessions of the experiment, and their data were included in the statistical analyses for repeated measures analysis of variance. Fifty of the participants were Czech students (28 female, 22 male; age range: 20–23) at Masaryk University, while twenty-five participants (15 female, 10 male; age range: 19–25) were American students at Columbia University. All students received one course credit for participating in the study. All participants had normal or normal-to-corrected vision, and none of them reported any memory pathologies. The concept set consisted of 48 concepts created for the purpose of the experiment. The concepts were two-word combinations of an adjective and a noun. To avoid any confounds caused by word frequency and length, we controlled for both factors. Nouns and adjectives were matched across concept categories for word length and frequency using the SUBLTLEX_us_ corpus database (available online). The SUBTLEX_us_ corpus (based on 51 million words) has been suggested as more suitable for psycholinguistic research than other popular corpus databases such as Celex or Brown corpus [54]. It was assembled from movies and television series subtitles. We used word frequency via SUBTLwf – an indicator of word frequency per million words.

The concepts comprising the different categories (CUL, ONT, INT) did not differ in terms of word frequency (target nouns: *F*(2,45) = .46, p = .64; target adjectives: *F*(2,45) = 1.37, p = .27) or word length (target nouns: *F*(2,45) = .34, p = .71; target adjectives: *F*(2,45) = .83, p = .45). For the American subset of subjects, individual concepts were presented in English, while Czech participants were presented with equivalent concepts translated into Czech. We used three independent translators, and only concepts in which they agreed 100% were used. The experiment was programmed in MATLAB (R2011b; MatWorks Inc., Natick, MA) and was carried out on a 17-inch color screen laptop. The viewing distance was approximately 50 cm.

Each concept could be classified according to the following attributes: *concept category*, *ontological category*, and *agent presence.* Each of the three concept categories consisted of 16 adjective-noun-pair concepts. In total, 48 concepts were presented. Within each concept category, 4 different ontological categories [22] were presented in the form of nouns (human, animal, plant, object); the adjective defined whether the concept was CUL (e.g., wooden bottle), ONT (e.g., evaporating rabbit), or INT (plastic clock). These four ontological categories defined the presence or absence of agents. Human and animal ontological categories incorporated a presence of agents, while plant and object categories represented the absence of agents. The list of individual concepts can be found in Table 1.

### Procedure

Prior to the experiment, subjects were told that the study involved learning concepts and their task would be to memorize all the presented concepts as their memory would be subsequently tested. The experiment consisted of three phases (study, distractor, and test) repeated twice in order to determine the pervasiveness of the ideas. During the study phase, the items were presented randomly at the center of the screen (white print on black screen) for 1500 ms with a 1000 ms interstimulus interval. A fixation cross was presented at the center of the screen prior to each concept presentation in order to indicate the beginning of a trial. The study phase was followed by a distractor task. Participants were given 2 minutes to solve simple mathematical operations.

In the final test phase of the experiment, a free recall test was presented. Subjects were prompted to recall all concepts, including both adjective and noun, by typing them individually on the computer screen. Correct recall required providing both components of the concept, i.e., adjective and noun. The recall was considered as accurate when both the adjective and the noun were recalled as shown originally during the test phase. No feedback was given during the free recall task. This sequence (the study phase, the distractor task, the test phase) was repeated in order to observe the pervasiveness of the concepts in memory (i.e. the effect of repeated exposure on recall of individual concepts).

## Results

A 3×2×2 (concept category [CUL, ONT, INT]×agent presence [present, absent]×test phase [immediate, delayed]) repeated-measures analysis of variance was computed in order to examine the effects of concept category and presence of agent presence on subsequent recall. There was a main effect of concept category: *F*(2,138) = 41.30, p<.001, η^2^_p_ = .37; and also a main effect of agent presence: *F*(1, 69) = 10.01, p<.01, η^2^_p_ = .13. Unsurprisingly, we found a main effect of test phase, F(1,69) = 79.79, p<.001, η^2^_p_ = . 54 which demonstrated that participants recalled significantly more information after the second test phase. An interaction between concept category and agent presence was also observed *F*(2,138) = 4.53, p<.05, with rather small effect size, η^2^_p_ = .07. Interactions between concept category and test phase, agent presence and test phase, and concept category, agent presence and test phase were not significant (p = .13; p = .41, p = .25, respectively).

**Figure 1:**
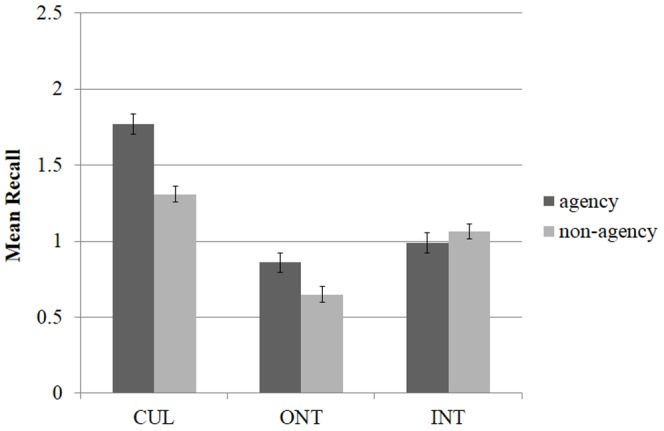
Mean recall in terms of concept category and presence of agents. Participants recalled significantly more agents involving concepts in cultural schema-level and domain-level breaches condition, however this was not true for intuitive concepts (without breaches). The figure represents data averaged for immediate Recall 1 and immediate Recall 2. Error bars represent standard errors.

As illustrated in Figure 1, in terms of the overall recall (both recall 1 and recall 2), follow-up t-tests using Bonferonni correction revealed that memory for CUL concepts (M = 6.21, SE = 0.28) was superior to both ONT (M = 3.28, SD = 0.28), *t*(69) = 8.71, p<.001, and INT concepts (M = 4.11, SE = 0.23), *t*(69) = 5.95, p<.001. Significant differences were observed between INT and ONT concepts, *t*(69) = −2.87, p<.01. These results (i.e., the immediate memory advantage for INT over ONT concepts) are in accordance with other studies [1], [5].

For both expectancy-violating concepts, agents-involving concepts were much better recalled than agents-devoid concepts. No difference in terms of the presence of agents was found for INT concepts. More specifically, for CUL concepts, concepts including agents were recalled to a greater extent (M = 3.59, SE = 0.25) than concepts devoid of agents (M = 2.63, SE = 0.17), *t*(69) = 2.94, p<.01. The same was true for ONT concepts: concepts comprised of agents had a memory advantage (M = 1.92, SE = 0.20), over those without agents (M = 1.32, SE = 0.14), (*t*(69) = 2.84, p<.01). Interestingly, for everyday INT concepts, recall did not differ in terms of agent presence (agents: M = 1.99, SE = 0.17; non-agents: M = 2.14, SE = 0.16), p = .50.

The two cultural (American and Czech) samples did not differ in terms of the observed results (p = .27; .48; .74 for differences in immediate memory recall 1 based on concept category, agent presence, and interaction between concept category and agent presence respectively; and p = .11; .70; .65 for differences in immediate memory recall 2 based on concept category, agent presence, and interaction between concept category and agent presence, respectively). In other words, both Americans and Czechs remembered agents and cultural schema-level information to a greater extent than non-agents, domain-level violations or intuitive information. Furthermore, both groups showed equal propensity toward remembering agents from expectancy-violating categories (cultural schema-level and domain-level), but memory for intuitive information was similar for both agents and non-agents.

## Delayed Recognition Memory

The first experiment revealed one of the factors that influence the memorability of concepts based on what kind of expectancy violation they possess. As has been suggested by previous research [1], [5], the success of cultural and religious ideas resides not in their immediate memory advantage, but rather in their memory advantage *over time*. Thus, we decided to test participants' memory of the concepts presented in Experiment 1 with a surprise recognition test. Initially, our pilot study demonstrated that recall after one month is highly impoverished, equal essentially to 1±1 concept for each participant. For this reason, we decided to use a recognition test. Participants were emailed approximately one month after the initial experimental phase with the request to fill out a brief follow-up questionnaire. They were asked to indicate which of the concepts they remembered seeing in the first part of the experiment. All items matched violation type and ontological categories of the original concepts.

### Method

The same subjects who took part in Experiment 1 participated in this study. Approximately one month (±2 days) after the initial test phase, subjects were contacted via email and invited to participate in the new study. The test included the initial 48 concepts and an equal number of new, fabricated concepts (included in the Appendix S1) that were semantically unrelated to any of the previous concepts (an equal number of fabricated concepts was created for each concept category in order to match the concepts previously presented). The participants' task was to determine which concepts were presented in the initial study by highlighting each of the concepts presented.

In total, 72 participants (of the original 75) completed the recognition test. Subjects received college credit for their participation.

### Results

**Figure 2:**
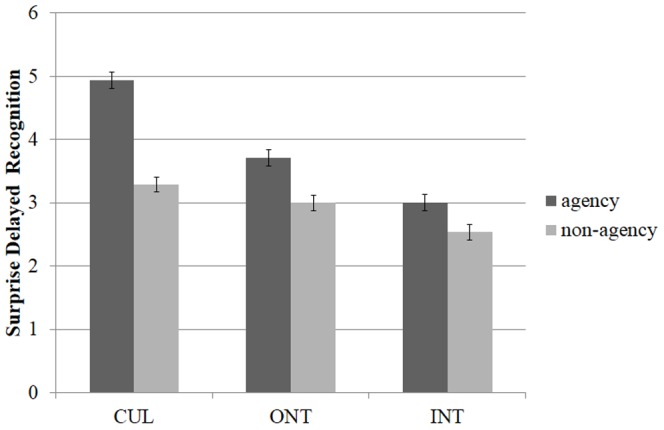
Surprise delayed recognition performance in terms of concept category and agent presence. Participants recalled significantly more agents-involving concepts in cultural schema-level and domain-level breaches condition; however this was not true for intuitive concepts (without breaches). Error bars represent standard errors.

A 3×2 (concept category×agent presence) repeated-measures analysis of variance showed that in terms of recognition, there was a significant main effect of concept category: *F*(2,142) = 33.15, p<.001, η^2^_p_ = .46; a significant main effect of agent presence: *F*(1,71) = 33.86, p<.001, η^2^_p_ = .32; and a significant interaction of concept category and agent presence: *F*(2, 142) = 10.99, p<.001, η^2^_p_ = .13. T-tests using Bonferroni correction showed significant differences between the recognition of CUL and ONT (t(71) = 4.78, p<.001), CUL and INT (t(71) = 7.72, p<.001), and ONT and INT (t(71) = 3.56, p<.01). The recognition of CUL concepts (M = 8.24, SE = 0.47) was superior to ONT concepts (M = 6.71, SE = 0.47) and INT concepts (M = 5.56, SE = 0.37). Furthermore, concepts including agents were recognized better than concepts without agents F(1,71) = 33.86, p<.001. Interestingly, the interaction of agent presence and concept category indicated that for CUL and ONT, agents-involving concepts were much better recognized than agents-devoid concepts (agents: M = 4.94; SE = 0.27; M = 3.71, SE = 0.26; non-agents: M = 3.29; SE = 0.25; M = 3.00, SE = 0.26, respectively). This was not true for INT concepts, where no differences between those conditions were found (agents: M = 3.01, SE = 0.22, non-agents: M = 2.54, SE = 0.23), even though a similar, but marginally significant trend was observed (p = .06). For further details, see Table 2 and Figure 2. The two cultural samples (American and Czech) did not differ in terms of the observed results (p = .816 for differences in the Recognition Test based on agent presence, and p = .725 for the interaction between concept category and agent presence respectively. Although there was a significant difference in terms of concept category (p = .014), the trend was still the same as in the previous experiment (Americans: M CAT = 4.17, M ONT = 2.83, M INT = 2.78; Czechs: M CAT = 10.14, M ONT = 8.53, M INT = 6.86).

**Table 2:** Means (standard error of means) for recall of individual concepts based on the presence of agents.

	Recall 1	Recall 2	Delayed Recognition
**Cultural schema-level expectancy-violating concepts**			
*Agents*	1.49 (0.15)	2.05 (0.15)	4.94 (0.27)
*Non-agents*	1.04 (0.11)	1.58 (0.12)	3.29 (0.25)
**Domain-level expectancy-violating concepts**			
*Agents*	0.72 (0.10)	1.20 (0.12)	3.71 (0.26)
*Non-agents*	0.60 (0.08)	0.70 (0.09)	3.00 (0.26)
**Intuitive concepts**			
*Agents*	0.72 (0.09)	1.26 (0.10)	3.01 (0.22)
*Non-agents*	0.74 (0.08)	1.39 (0.12)	2.54 (0.23)

*Note. N* = 75 (Recall 1, Recall 2), *N* = 72 (Delayed recognition).

## Discussion

Our study included stimuli involving cultural schema-level, and ontological (domain-level) expectation violations, and intuitive information (ideas without any violation). We used immediate recall and surprise delayed recognition tasks (one month after the initial test phase) in order to examine which ideas enjoy memory advantages and their attractiveness to our cognitive architecture. Our findings suggest that although violations of ontological intuitions may confer mnemonic advantages, violations at the category level are consistently more powerful in creating even long-term memory effects. Importantly, the presence of agents plays a crucial role in attracting attention, as shown by the higher memorability of agents-including concepts across categories.

In Experiment 1 the main interest was to see the effect of expectation violation type (domain versus cultural schema-level expectation violation, or no violation) as well as the effect of agent presence on immediate recall. Both immediate recall tasks showed that information that includes cultural schema-level violations is recalled more easily than information with domain-level violations or without any expectation violation. However, the second recall test phase demonstrated better recall for both kinds of expectation-violating information. In the surprise delayed recognition test that took place one month after the initial learning phase we aimed to examine the long-term memory effects and resistance toward forgetting for individual concepts. The two sets of results are fairly consistent with the previous findings, and together they are in accordance with proposed hypotheses about the superior memorability of minimally counter-intuitive ideas over time [5].

These results are not very surprising by themselves. In psychology, the term “distinctiveness effect” refers precisely to this enhanced memory for distinct events [10], [11], [12], [13], [15]– in our case expectation-violating ideas. Enhanced memory for information including expectation violations has been documented in many studies [14], [45], [47]. Increased processing of expectation-violating information has also been reflected in electrophysiological studies of social perception [47], concepts embedded in sentences [37], face perception [48], or unexpected events embedded in array of expected stimuli [49]. Moreover, it was shown that items from weakly related words (i.e., unexpected) are recalled better than strongly related word pairs, the phenomenon dubbed the “expectation-violating effect” [46]. Based on this evidence, it seems that expectancy-violating concepts create a special encoding in our memory, and thus are recalled easier and to a greater extent than intuitive ones.

Our results provide an important addition to the state of the art in research on concept memorability, showing that ideas violating attributes to which we are more conditioned (i.e., culturally familiar, and thus more likely to occur) are more attractive to our cognitive architecture compared with ideas that violate ontological expectations (i.e. those that cannot occur in real life). This finding might have important evolutionary implications regarding the role and importance of cultural conditioning in human cognition. Our brains retain more information about unexpected and unpredictable changes in the environment and assign less attention to familiar stimuli, and cultural environments give specific forms to this tendency by habituating the mind to certain stimuli more than others.

Of course, certain types of stimuli, agents being one of them, are cross-culturally salient. Any kind of intentional agent in our environment is a potential object of interaction, be it a threat or an opportunity [39]. Thus, when presented with expectancy violations that pertain to agents, attention seems to be attracted to this type of information more than for intuitive, everyday agents or for expectancy violations that pertain to non-agents. Pascal Boyer [50] has identified intuitive ontological domains that are cognitive adaptations used to make sense the various entities in our world. Those intuitive ontologies (intuitive psychology, biology, and physics) represent persons, animals, plants, and objects. The most distinct aspect that separates persons and animals from plants and objects as ontological domains is the presence of intentionality and the likely attribution of mental states to the former. This capacity of attribution of intentionality to animate beings develops as early as 15 months of age [51], while the understanding of self-propulsion of animate objects develops at around 5 months of age [52]. This bias toward agency detection leads to what has been termed the “hyperactive agency detection device” (HADD) [40]; that is a tendency to over-attribute agency to ambiguous stimuli in our environment [39]. Thus, the presence of agents that violate our expectations seems to be a particularly salient feature in attracting attention; these agents might trigger sensitivities related to predation, survival, threat, reputation-management, and so on.

Our results add an important nuance to these findings. Intuitive, mundane ideas are treated by our cognitive systems as equally attractive and attention-grabbing regardless of agent presence (whether it is a human or a plant). However, ideas that violate our expectations (either domain- or cultural schema-level) in a mild fashion are more memorable if they pertain to agents (humans and animals) than to non-agents (plants or objects/artifacts. Importantly, this is the case even for objects and plants that acquire agent-like qualities, i.e., minimally counterintuitive concepts. Irrespectively, however, the combination of schema-inconsistent or expectancy-violating information and agents seems to provide a cognitive optimum for the memorability of ideas, and by extension the potential transmittability of these concepts.

These results have certain limitations. In order to achieve the high level of control provided by the laboratory, one always loses some relevance and ecological validity. For example, equal processing times do not occur in real life, where individuals can allot as much attention as they require to particular stimuli, creating various associations or narratives in their mind. Importantly, however, research has demonstrated that the enhanced memory for distinct events (“the distinctiveness effect”) is not modulated by processing time [53].

Furthermore, in the real world, people are not explicitly asked to remember any fixed set of concepts. Each concept competes freely and interacts with an enormous number of other concepts, and among a host of ecological and social factors that play crucial roles in their “natural” transmission. While this is true, and we hope further research will investigate the roles of other factors than the character of the information in memory (such as source memory), our study still provides important insights on the organization of information in semantic memory by examining memorability in the sense of “all else being equal”.

Finally, our study only speaks to the memorability of *concepts*. It is likely that when it comes to actual entities, domain-level violations would be more salient that cultural schema-level ones (actually encountering a talking tree would probably be more memorable than encountering a wooden bottle). On the other hand, some concepts (for example religious ones) consist precisely of impossible ideas that are not encountered in the real world; although no one has witnessed a resurrection, memes regarding beings who rise from the dead can often be very successful. Moreover, imagery levels modulate the memorability power, though minimally counter-intuitive concepts are not affected by the extent to which the idea is imaginable [55]. Future studies could explore the additional effects of the effect of viability (how likely an idea is to occur), imagery, and social factors in the memorability of ideas.

Overall, our results reveal some of the complexities of semantic memory, in particular some of the various parameters that affect the successful encoding of concepts [50]. Two such parameters are expectation-violation and the presence of agents. In particular, the combination of those two conditions places concepts in an optimal position for attracting attention and making ideas cognitively appealing and potentially better suited for cultural transmission.

## Supporting Information

Appendix S1 **Fabricated concepts used in the recognition task.**
(DOCX)
