# Supplementary material for: Memory for Expectation-Violating Concepts: The Effects of Agents and Cultural Familiarity
Source: PLoS One. 2014 Apr 8;9(4):e90684. doi: 10.1371/journal.pone.0090684 (PMC3979650; doi:10.1371/journal.pone.0090684)
Supplement: Appendix S1 — Fabricated concepts used in the recognition task. (DOCX) [file pone.0090684.s001.docx]

altruistic banker

big fish

black bean

burping pie

colonial garden

cubical worm

diligent tailor

disappearing painter

errorprone editor

fast swimmer

featherless bird

flying moth

glass kitchen

glass pumpkin

good baker

graceful glass

immoral judge

invisible flower

invisible lettuce

invisible zebra

metal melon

metal notebook

mild pepper

obese model

offended giraffe

omnipotent actor

omnipresent scientist

paper window

partying lizard

praying wine

rotten tomato

running doors

Russian parrot

seedless corn

singing gecko

skinless apple

small potato

small window

smart book

smiling lily

soaking oak

stinging bee

sweet pepper

swimming cod

twolegged turtle

white flower

winking lamp

yellow salami
